# Low-Level Laser Therapy in Maxillofacial Trauma: A Prospective Single-Arm Observational Study

**DOI:** 10.3390/dj13110532

**Published:** 2025-11-13

**Authors:** Raissa Dias Fares, Jonathan Ribeiro da Silva, Sylvio Luiz Costa De-Moraes, Jose Mauro Granjeiro, Monica Diuana Calasans-Maia

**Affiliations:** 1 Post-Graduation Program in Dentistry, Fluminense Federal University (UFF), Niteroi 24220-140, RJ, Brazil; raissafares@id.uff.br; 2 Department of Oral and Maxillofacial Surgery, University Center Serra dos Orgãos (UNIFESO), Teresopolis 25964-004, RJ, Brazil; 3 Clinical Research Laboratory, Dentistry School, Fluminense Federal University, Niteroi 24220-140, RJ, Brazil

**Keywords:** low-level light therapy, paresthesia, maxillofacial injuries, nerve regeneration, infraorbital nerve, inferior alveolar nerve, mental nerve

## Abstract

**Background:** Surgical management of trauma in the maxillofacial complex can result in iatrogenic nerve injuries, particularly involving the infraorbital, inferior alveolar, and mental nerves. Paresthesia is a common postoperative complication, often attributed to the anatomical positioning of these nerve structures, making them vulnerable to injury. Among current therapeutic options for nerve injuries, low-level laser therapy (LLLT) has shown promising results in published studies. **Objectives:** This prospective observational study evaluated the effects of LLLT on nerve recovery following maxillofacial trauma surgery. **Methods:** A total of 21 participants, with a median age of 35 years and no gender-based selection criteria, were enrolled. Cases included zygomaticomaxillary complex and mandibular osteosynthesis; analyses were within-subject across time. Postoperative laser therapy was administered to both groups using the DUO MMO device (MMOptics, São Carlos, Brazil), delivering infrared light along the pathways of the inferior alveolar, infraorbital, and mental nerves. Nerve function was assessed regularly using a Visual Analog Scale (VAS) and the Brush Stroke Direction (BSD) test to evaluate sensory recovery. **Results:** Compared with baseline (15 days post-op, pre-LLLT), VAS scores showed significant reductions at sessions 7 and 10, and BSD responses increased over time. **Conclusion:** After multiplicity control, only the session 10 comparison remained significant. These observational findings support the feasibility of multi-session LLLT after maxillofacial trauma; controlled trials are warranted to determine efficacy.

## 1. Introduction

Maxillofacial trauma is a frequent occurrence in emergency and trauma centers, often involving complex fractures that require surgical intervention to restore facial function and aesthetics [1,2]. Surgical exposure and fixation are essential for adequate reduction in facial fractures; however, due to the dense innervation of the maxillofacial region, these injuries are frequently accompanied by sensory disturbances [3,4].

Importantly, in patients with maxillofacial fractures, neurosensory impairment is usually present prior to surgical management, resulting directly from the traumatic insult to the peripheral branches of the trigeminal nerve. Such deficits commonly affect the infraorbital, mental, and inferior alveolar nerves, leading to paresthesia, dysesthesia, or hypoesthesia [5,6,7]. These symptoms significantly affect patients’ quality of life, as the oral and perioral regions have one of the highest concentrations of sensory receptors in the body.

Although peripheral nerves possess an intrinsic capacity for regeneration, spontaneous recovery is often incomplete and varies according to the extent of injury, patient age, and time elapsed before intervention [8,9]. Persistent neurosensory disturbances are therefore a common sequela of facial fractures, even after successful surgical repair. This highlights the need for adjuvant therapies capable of enhancing neural recovery and improving sensory outcomes.

Low-level laser therapy (LLLT), also known as photobiomodulation, has been proposed as a noninvasive modality to promote neural regeneration and functional recovery. LLLT is believed to stimulate mitochondrial activity, improve local microcirculation, and modulate inflammatory responses, resulting in enhanced tissue repair and nerve function [10,11,12]. Previous studies have suggested beneficial effects of LLLT in various peripheral nerve injuries; however, evidence specifically addressing post-traumatic sensory deficits in maxillofacial fracture patients remains limited.

Therefore, this prospective single-arm observational study aimed to evaluate the effects of postoperative LLLT on the recovery of trauma-induced neurosensory disturbances in patients presenting with midfacial or mandibular paresthesia following surgical management of facial fractures.

## 2. Materials and Methods

This study complied with the principles outlined in the Declaration of Helsinki and was conducted in accordance with CNS Resolution 466/2012. Ethical approval was obtained under protocol number 6.464.607, 26 October 2023. This study is registered in the Brazilian Registry of Clinical Trials (RBR-98xmt2n).

This study design, conduct, and reporting adhered to the STROBE (Strengthening the Reporting of Observational Studies in Epidemiology) guidelines [13].

Sample size was estimated for a paired continuous outcome (change in VAS from baseline to session 10) using G*Power (Heinrich Heine Universität Düsseldorf, v3.1.9.6 macOS) (paired *t*-test, two-tailed, α = 0.05, power = 0.80). Based on pilot data from the present cohort (*n* = 18 pairs), we assumed a mean within-subject change of Δ = 4.50 VAS units with a standard deviation of the paired differences SD = 2.83 (effect size dz = 1.59), yielding a minimum of *n*= 4 participants. Allowing for approximately 5% attrition, the practical target was set above this value; our final target remained *n* = 20 for feasibility and precision.

A total of 21 participants requiring surgical management of their traumatic injury were recruited from the Maxillofacial Surgery Service at Hospital das Clínicas de Teresópolis (HCTCO) between August 2022 and May 2024. Eligibility criteria were independent of sex, age, or race, and all participants signed an Informed Consent Form (ICF) prior to participation. Surgical procedures were performed by the same experienced maxillofacial trauma team to ensure consistency across interventions.

The selection criteria included participants requiring open reduction and internal fixation for either orbital-zygomatic-maxillary fractures (Group 1 *n* = 12) or mandibular body fractures (Group 2 *n* = 9), as these injuries allowed for the presence of an uninjured side for comparative sensory assessment. All analyses were within-subject across time; no between-group comparisons were planned.

In Group 1, both extraoral and intraoral surgical approaches were employed, whereas Group 2 was managed exclusively through intraoral access. Although the fracture sites were classified as simple, either non-displaced or comminuted, patients in both groups exhibited some degree of paresthesia before surgical intervention.

All patients received the same oral medication regimen after surgical management, consisting of amoxicillin for seven days, along with nimesulide and dipyrone for three days.

Participants presenting any degree of sensory alteration in the midface or mandible after the surgical management were assessed 15 days postoperatively, following the initial healing period and resolution of postoperative edema.

Early initiation of low-level laser therapy (LLLT) before spontaneous nerve recovery and sufficient edema reduction was avoided, as residual swelling could interfere with laser beam penetration, potentially diminishing the effectiveness of the treatment for paresthesia.

Participants who did not require surgical intervention, pregnant women, individuals undergoing orthognathic surgery, those with panfacial fractures, or displaced or comminuted fractures were not eligible. Participants with a history of facial surgery or bilateral fractures, which prevented comparative sensory assessment, were excluded.

LLLT has not been associated with worsening of sensory deficits in prior reports. Therefore, any unsatisfactory outcomes observed during this study were considered a continuation of pre-existing sensory impairment rather than a consequence of the intervention.

The LLLT protocol was determined by several factors, including the wavelength of the light, power output, duration, and frequency of irradiation. Participants received two laser therapy sessions per week, beginning 15 days postoperatively, for a total of ten sessions.

Participants were treated with a DUO MMO laser (MMOptics, São Carlos, Brazil) emitting infrared light at a wavelength of 808 nm along the pathways of the inferior alveolar, infraorbital and mental nerves (Table 1). The laser parameters applied were 100 mW of power, 3.6 W/cm^2^ of irradiance, and 2 J of energy per point, with an energy density of 66.7 J/cm^2^. Each point was exposed for 20 s, with a 1 cm distance between irradiation points (Table 1).

**Figure 1:**
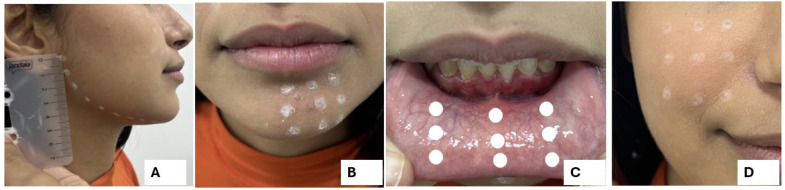
Irradiation Point Distribution for Nerve Pathways in Maxillofacial Trauma Treatment. (**A**) Irradiation points spaced 1.0 cm apart in focused (contact) mode along the inferior alveolar nerve pathway, from the mandibular angle to the chin. Linear irradiation points were applied along the middle third of the mandible (single row), following the mandibular canal. (**B**) Irradiation points spaced 1.0 cm apart in focused (contact) mode for the mental nerve, covering the region between the lower lip, the base of the mandible (edge of the chin), and the location of the mental foramen. If involvement was unilateral, irradiation was extended to the midline region. (**C**) Intraoral irradiation of the lower labial mucosa for the mental nerve, with points distributed over the affected area. (**D**) Irradiation points spaced 1.0 cm apart in focused (contact) mode along the infraorbital nerve pathway, covering the region limited by the lower eyelid, nasal ala, upper lip, and cheek.

Each treatment area received nine irradiation points, marked using a Denmark white dermographic pen, number 1440 (Viscot Medical LLC, East Hanover, NJ, USA), with markings removed following the laser session (Figure 1). Throughout the treatment, the laser tip was maintained firmly in direct contact with the skin, perpendicular to the treatment surface, and care was taken to ensure the area was clean and free of interference, such as makeup and facial hair (Figure 1).

Sensory assessments were carried out during the first, fourth, seventh, and tenth sessions. Subjective sensory evaluation was performed using a Visual Analog Scale (VAS). Objective clinical neurosensory testing involved the brush stroke direction (BSD) test, performed at a controlled velocity of approximately 2–3 cm/s.

Preoperative objective and subjective sensory analyses were not performed, as the participants were expected to undergo significant surgical manipulation, which could independently affect sensory responses.

### 2.1. Data Collection

Sensory assessments were conducted using both subjective and objective measures. Subjective evaluation was performed with the VAS, while objective assessment was conducted using the BSD test. Each participant completed the VAS 15 days after the surgical intervention and prior to each of the ten laser therapy sessions. The VAS consists of a 10 cm straight line with numerical labels from 0 to 10, where 0 indicates the absence of sensory change and 10 represents complete sensory alteration. Participants were instructed to select the value that best represented their current sensory change.

The VAS scores were subsequently reassessed during the fourth, seventh, and tenth sessions to monitor sensory recovery or persistence of impairment. The objective of the BSD test was to assess mechanoreceptor function through tactile stimulation. This test evaluates the participant’s ability to perceive contact at two nearby points or to describe the direction of a stimulus. The assessment was conducted using a #6 brush applied with consistent touch/pressure in the infraorbital, lower lip, and external mental regions.

All assessments were conducted in a well-lit, quiet, and comfortable environment. Each test was clearly explained and demonstrated before evaluation. For sensory testing, the contralateral (non-operated) side served as a within-subject reference (not a separate control group). Baseline was defined as the visit 15 days post-operation (pre-LLLT). Both the VAS and BSD were administered individually by the same operator in a consistent sequence.

### 2.2. Statistical Analysis

Repeated VAS measurements across four sessions (S1, S4, S7, S10) were analyzed with the Friedman test. Effect size was summarized by Kendall’s W. To localize differences while controlling family-wise error, we used Dunn’s multiple comparisons test (Prism default) after a significant Friedman result.

Binary outcome (BSD). The binary BSD across the same four sessions was first assessed with Cochran’s Q (complete cases only). If significant, we performed pairwise McNemar tests (exact binomial p when appropriate) for three prespecified contrasts versus baseline (S1 vs. S4, S7, S10), with Holm adjustment for multiple testing.

Missing data. Global tests (Friedman; Cochran’s Q) used complete cases across all four sessions. Pairwise tests (Dunn; McNemar) used pairwise deletion (all participants with data at the two time-points). No imputation was performed.

Software. Analyses were conducted in GraphPad Prism (v10.x). Two-tailed α = 0.05.

## 3. Results

**Figure 2:**
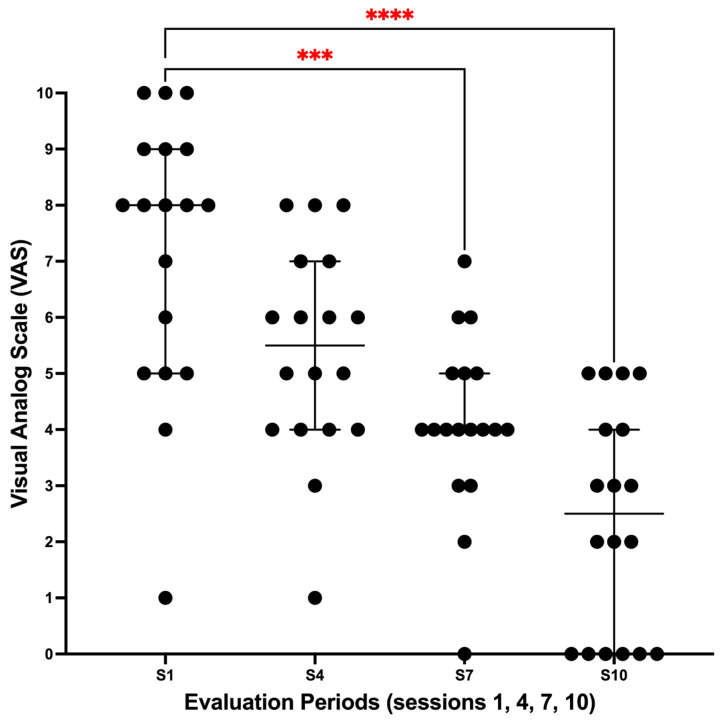
Visual Analog Scale (VAS) across sessions (session 1 = baseline; sessions 4, 7, and 10). Dots represent individual participant values; bars indicate the median and 95% confidence interval. Friedman χ^2^(3) = 45.35, *p* < 0.0001; Kendall’s W = 0.84. Dunn’s multiple comparisons versus baseline: session 4, *p* = 0.158; session 7, *p* = 0.0001; session 10, *p* < 0.0001. The red asterisks (*** and ****) indicate the statistical significance level.

A total of 21 participants were enrolled in the study and underwent post-surgical treatments and evaluations. Demographic analysis revealed a broad age range from 17 to 62 years, with a median age of 35 years and a standard deviation (SD) of 14.8 years (Figure 2). Regarding gender distribution, the cohort consisted of 15 men and 6 women. Sensory evaluations using the VAS and BSD tests were conducted at four distinct time points. However, two participants did not return for follow-up assessments, and one participant only completed the first and second evaluations.

The VAS analysis showed a progressive within-subject decrease over time, with significant reductions emerging from the third and fourth evaluation periods (sessions 7 and 10) relative to baseline (Figure 2). Eighteen participants contributed complete cases. VAS scores varied across sessions (Friedman χ^2^(3) = 45.35, *p* < 0.0001), with a large effect size (Kendall’s W = 0.84). Multiple comparisons (Dunn, adjusted) showed no difference between evaluation period 1 (session 1, baseline) and period 2 (session 4; adjusted *p* = 0.158), whereas significant reductions were observed at period 3 (session 7; adjusted *p* = 0.0001) and period 4 (session 10; adjusted *p* < 0.0001).

**Figure 3:**
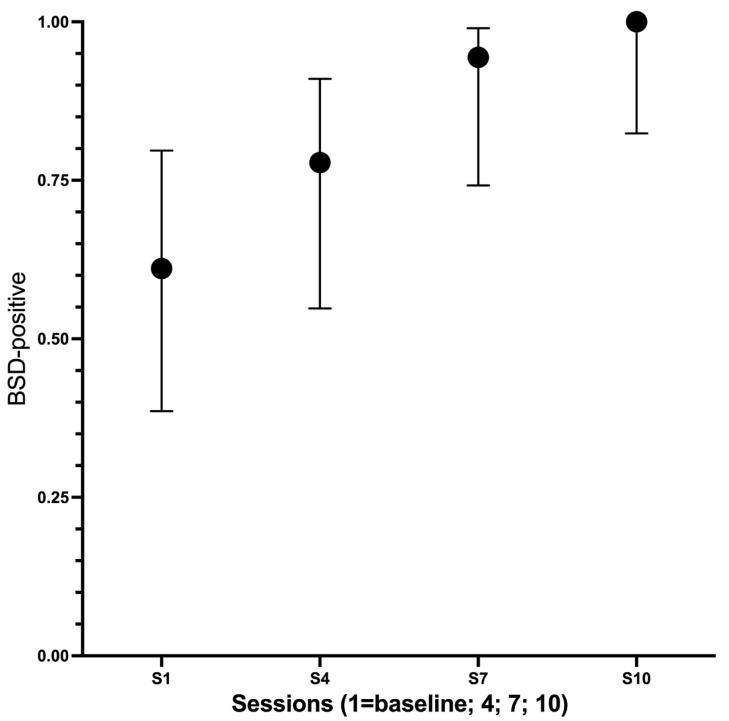
Binary sensory discrimination (BSD) across the four evaluation periods (session 1 = baseline; sessions 4, 7, and 10). Points show the proportion BSD-positive with 95% binomial confidence intervals (Wilson). Complete-case denominators *n* = 18. Cochran’s Q = 15.0, df = 3, *p* = 0.0018. Pairwise McNemar tests versus baseline with Holm adjustment: session 4, *p* = 0.25 (discordant b/c = 0/3); session 7, *p* = 0.0625 (0/6); session 10, *p* = 0.0469 (0/7).

Among the 21 enrolled participants, 12 (57%) were BSD-positive at baseline. Raw counts increased to 15, 17, and 18 by sessions 4, 7, and 10, respectively (Figure 3). For the global analysis, two participants had no BSD assessments, and one had partial data (session 1/session 4 only); therefore, 18 complete cases were analyzed. In this set, BSD positives rose from 11/18 (61.1%) at baseline to 14/18 (77.8%) at session 4, 17/18 (94.4%) at session 7, and 18/18 (100%) at session 10. Cochran’s Q indicated a global change across sessions (Q = 15.0, df = 3, *p* = 0.0018). Pairwise McNemar tests versus baseline, Holm-adjusted across three prespecified contrasts, yielded p_adj = 0.25 for session 1 vs. 4 (discordant b/c = 0/3), p_adj = 0.0625 for session 1 vs. 7 (0/6), and p_adj = 0.0469 for session 1 vs. 10 (0/7), indicating a monotonic increase with a statistically significant change from baseline to session 10 after multiplicity control.

Participants’ ages ranged from 17 to 62 years (*n* = 21). The median age was 35 years, with an interquartile range of 25 to 52.5 years; the 95% confidence interval for the median was 25 to 50 years. For completeness, the mean age was 37.5 years (SD 14.8).

## 4. Discussion

The development of neurosensory disturbances remains a significant concern for surgeons following maxillofacial trauma repair. Surgical procedures inevitably induce swelling during the tissue healing process, which can compress nearby nerves and lead to functional impairment [14]. In the most favorable scenarios, the resolution of inflammatory responses restores normal nerve function. However, nerve fibers are often subjected to additional traction and compression during surgery, which can result in varying degrees of neurosensory dysfunction.

Paresthesia is inherently unpredictable. LLLT was initiated 15 days postoperatively to allow spontaneous edema-related paresthesia to regress; this timing is consistent with clinical observations up to day 15 [15].

VAS is a well-established tool for assessing subjective experiences along a continuum [16]. This study provided a practical method to track sensory disturbances over time. Our use of VAS aligns with Poort et al., who supported its application for monitoring inferior alveolar nerve injuries [17], and with Miloro et al., who used VAS to follow subjective neurosensory changes after sagittal ramus osteotomy [18].

The wavelength range for LLLT commonly reported in the literature spans 660–820 nm [19,20]. We used 808 nm based on reports describing favorable outcomes for inferior alveolar nerve conditions at this wavelength [21,22,23,24]. Our findings align with those of Khullar et al. and Gianni et al., both of whom maintained a gender-neutral approach, ensuring no bias based on gender in their methodologies [19,20].

Regarding treatment frequency, we scheduled LLLT twice weekly over five weeks. This choice was guided by reports in maxillofacial and orofacial contexts that used multi-session, twice-weekly protocols and described favorable neurosensory or pain trajectories, while also balancing feasibility and adherence [22,23].

Prior studies applying near-infrared LLLT (for example, 808 nm at low power, 2–3 J per point) reported improvements across sessions, particularly when treatment was initiated earlier in the postoperative period [24]. To minimize early edema effects and to align with this literature, we began LLLT 15 days after surgery and used that visit as the analytical baseline. In our cohort, temporal patterns observed in VAS and BSD were compatible with gradual change across sessions; the single-arm design, however, precludes causal inference about timing or dose.

The BSD test was previously performed by Miloro et al., Khullar et al., and Bath et al. to objectively assess neurosensory function in patients [17,18,20,25]. In our study, BSD proportions increased over time, approaching near-normal values by the third and fourth evaluations, with a Holm-adjusted significant gain versus baseline only at session 10.

Using nonparametric repeated-measures methods, we observed consistent within-subject reductions in VAS across sessions (Friedman χ^2^(3) = 45.35, *p* < 0.0001; Kendall’s W = 0.84), with clinically evident change from the third evaluation onward. For BSD, proportions increased globally over time (Cochran’s Q = 15.0, df = 3, *p* = 0.0018); pairwise tests versus baseline, adjusted by Holm across three prespecified contrasts, confirmed a significant gain only at session 10. These temporal associations support a monotonic improvement during the multi-session protocol, while causal inference awaits controlled trials. Given the single-arm design, these findings should be interpreted as associations rather than proof of efficacy. Missingness was handled conservatively (complete cases for global tests; pairwise deletion for contrasts), and results were consistent across available data.

The mechanism of action of LLLT is believed to involve photon absorption by cellular chromophores, modulation of the mitochondrial electron transport chain, and downstream signaling that may support tissue repair [26]. Responses can vary with the baseline redox state, which may influence mitochondrial retrograde pathways beyond ATP production alone [27]. Such context dependence may explain variability across cell types and the occasional absence of measurable clinical effects [26]. The principal therapeutic properties described for LLLT include anti-inflammatory, analgesic, and pro-repair effects, and the method is simple and cost-effective, allowing use as an adjunct or, in selected settings, as a stand-alone approach [28]. Further, the methodology is simple, cost-effective, and can be integrated as an adjunct to conventional treatments or as a stand-alone alternative for certain conditions.

Most studies to date have focused on planned osteotomies, such as third-molar removal and orthognathic surgery. In contrast, our findings highlight potential utility after trauma surgery. The observed trajectories in this cohort suggest that LLLT may be a promising component of postoperative management in maxillofacial trauma and reconstruction, warranting confirmation in controlled trials.

## 5. Conclusions

In this single-arm cohort, VAS scores decreased over time, and BSD positives increased across sessions. Reductions in VAS were evident at the third and fourth evaluation periods (sessions 7 and 10). For BSD, the only Holm-adjusted pairwise difference versus baseline occurred at session 10. These findings indicate temporal associations compatible with progressive neurosensory improvement during a multi-session LLLT protocol. Controlled studies are required to determine efficacy, define optimal parameters, and explore whether specific nerve distributions respond differentially. In addition, low-level laser therapy (LLLT) may be combined with agents such as vitamin B, pentoxifylline, or galantamine to enhance therapeutic outcomes.

### Limitations

This study lacks a control group and masking, which limits causal inference and increases the possibility that spontaneous recovery contributed to the observed changes. The follow-up covered only the treatment phase, precluding assessment of long-term durability. Missing measurements led us to use complete cases for global tests and pairwise deletion for contrasts; although results were consistent, the modest sample size reduces precision. Repeated BSD testing may also introduce practice effects. These limitations underscore the need for adequately powered, controlled trials to clarify the role of LLLT in post-trauma neurosensory management.

## Figures and Tables

**Table 1:** Description of Irradiation Points for Nerve Pathways in Maxillofacial Trauma Treatment. Adapted from Oliveira et al., 2015 [10].

Nerve	Extraoral	Intraoral
**Mental**	Irradiation points spaced 1.0 cm apart in focused (contact) mode. The region between the lower lip, the mandibular base, and the mental foramen was irradiated. If the involvement was unilateral, irradiation extended to the midline (Figure 1B).	Irradiation points spaced 1.0 cm apart in focused (contact) mode, targeting the lower lip mucosa with points distributed over the affected area (Figure 1C).
**Infraorbital**	Maxillary irradiation points spaced 1.0 cm apart in focused (contact) mode. The region between the lower eyelid, alar nose, upper lip, and cheek was irradiated (Figure 1D).	Not performed.
**Inferior Alveolar**	Irradiation points spaced 1.0 cm apart in focused (contact) mode, covering the region from the mandibular angle to the chin. Linear irradiation points were applied along the middle third of the mandible (single row), following the mandibular canal (Figure 1A).	Not performed.

## Data Availability

The data presented in this study are available on request from the corresponding author due to ethical restrictions.
